# Children with disabilities in Safe Community 

**Published:** 2019-08

**Authors:** Miodrag Blizanac, Mirjana Milankov, Jovan Vignjevic

**Affiliations:** ^*a*^Nongovernmental organization Faith, Love, Hope, Novi Sad, Serbia.; ^*b*^National center for injury prevention and safety promotion, Novi Sad, Serbia.

## Abstract

Care about people with disabilities is very important for every community, special for safe community. In Safe Community Novi Sad works Nongovernmental organization Faith, Love, Hope. This organization was founded on March 2010. To promote the idea of social inclusion. The main goal of the founders was to organize free time for disabled children out of their school frame. These activities also include the children’s parents, friends and people from the neighborhood, as well as all others willing to help working with children with different kinds of handicap. Organization gathers more than 100 members, mainly disabled children. The majority of these kids attend the special school “Milan Petrović“ as well as children of different ages from the other ordinary schools, where they all have equal chances to develop their potentials and prepare for mutual collective events. Faith, Love, Hope has following activities: mixed inclusive choir ISON, Theater of good vibrations, section for fisherman and environmental care, section for activities with the animals, orientation, dance and folklore dance, section for photography as well as other opportunities for children to develop different skills and open new possibilities for improving their physical and mental evolution. The crucial aims concerning disabled children are of supportive kind: by putting them into social position with equal chances. Faith, Love, Hope strengthen their spiritual and mental level. Through this kind of motivation the whole society should built much more responsible relation with disabled children and in general, handicap people. Due to that course, this organization intensifies activities through the form of concerts, projects, organizing travels and different kind of promotions. The main activity of our organization is organization of free time for children with disabilities and persons with disabilities. We are organizers of International Inclusive festival “Hearts in Harmony” for the fifth year in a row. The festival gathers inclusive choirs and vocal groups from all over Europe. The Inclusive choral workshops and festival are the best indicators of inclusion in practice. For five days people with disabilities together demonstrate their achievements by singing in the streets of host cities. This event is of great importance both for children with disabilities and development disorders and for all those who found joy in singing with our children as well for entire community music and positive vibrations are moving the whole city. Within our association we formed a section for activities with horses. This is a very important aspect of activities for our children that affect their mental, physical and emotional development. Besides caring for the horses, maintain their hygiene, feeding, etc. children with help of trained instructors perform exercises on horseback. To succeed they have to engage all the muscles and senses, control the head and body, their cognition develops and their self-esteem grows. For someone who is disabled or limited in movement, it’s a thrill when you start a big horse and force him to do what you want, and that is to move. Success in this is a real injection of confidence. Therapeutic riding is a special type of rehabilitation program that includes pedagogical, psychological, psycho therapeutic, rehabilitative and social - integrative effects, using spec training and trained horses. Equestrian Club Filipus carries out sports activities and provides the conditions for dealing with equestrian sports for club members and interested citizens. Horseback riding, hippotherapy and riding para- dressage will be organized within the club. The club will be focused on dealing with sports work with people with disabilities. Our wish is that the founders of the Club, persons with disabilities, are encouraged to creative thinking and sports but also other skills and independence and to open up new opportunities to improve their mental and physical development. Mission the Good Vibrators Theater is to promote the positive achievements of children with disabilities, young people and people with disabilities in order to integrate them into the social life of the community. The goal is to build a society where there are places for each individual. In this unusual theater, there are heart rings that vibrate together and unite in the basic thread - that we are all equal, that we have the same rights to expression and expression of skill, talent and effort. As part of our association, on our farm Filipus we have free space where we want to produce soda (carbonated water) and juices (klaker and kabeza) and to employ single parents who are members of our association and children with disabilities who can work in the production process (arrange bottles, stick labels, etc.).

**Keywords:**

Safe Community, People with disabilities, Choir, Theater, Social Enterprise, Equestrian Club

